# Multifrequency synthesis and extraction using square wave projection patterns for quantitative tissue imaging

**DOI:** 10.1117/1.JBO.20.11.116005

**Published:** 2015-11-02

**Authors:** Kyle P. Nadeau, Tyler B. Rice, Anthony J. Durkin, Bruce J. Tromberg

**Affiliations:** a Beckman Laser Institute, Laser Microbeam and Medical Program, 1002 Health Sciences Road, Irvine, California 92612 United States; b Beckman Laser Institute, Laser Associated Sciences, Photonic Incubator, 1002 Health Sciences Road, Irvine, California 92612 United States

**Keywords:** structural illumination, diffuse optical spectroscopy, wide-field imaging, image processing, signal processing, digital micromirror device

## Abstract

We present a method for spatial frequency domain data acquisition utilizing a multifrequency synthesis and extraction (MSE) method and binary square wave projection patterns. By illuminating a sample with square wave patterns, multiple spatial frequency components are simultaneously attenuated and can be extracted to determine optical property and depth information. Additionally, binary patterns are projected faster than sinusoids typically used in spatial frequency domain imaging (SFDI), allowing for short (millisecond or less) camera exposure times, and data acquisition speeds an order of magnitude or more greater than conventional SFDI. In cases where sensitivity to superficial layers or scattering is important, the fundamental component from higher frequency square wave patterns can be used. When probing deeper layers, the fundamental and harmonic components from lower frequency square wave patterns can be used. We compared optical property and depth penetration results extracted using square waves to those obtained using sinusoidal patterns on an *in vivo* human forearm and absorbing tube phantom, respectively. Absorption and reduced scattering coefficient values agree with conventional SFDI to within 1% using both high frequency (fundamental) and low frequency (fundamental and harmonic) spatial frequencies. Depth penetration reflectance values also agree to within 1% of conventional SFDI.

## 1 Introduction

Techniques that analyze light propagation in the spatial frequency domain with structured illumination patterns can be used for quantitative characterization and imaging of biological tissue.^1^ The relationship that governs this approach is known as the spatial modulation transfer function (s-MTF). The s-MTF describes the dependence of the attenuation of spatial photon density waves in turbid media in terms of the spatial frequency of illumination and the absorption and scattering properties of the sample. Dognitz and Wagnières^2^ reported the first use of spatial frequency domain methods for measuring tissue optical properties (i.e., absorption and reduced scattering coefficients). The authors employed a radially-varying square wave illumination pattern, applying one-dimensional (1-D) Fourier transforms to a cross-section of the pattern, and utilized the intensity value corresponding to the DC (planar illumination) and fundamental frequency components. In this case, optical properties were determined at a single point in space. Bevilacqua et al.^3^ developed an alternate method using two-dimensional (2-D) spatial frequency domain analysis of dot patterns resulting in the mapping of the sum of spatial frequency components in the s-MTF over a frequency band, and the derivation of the spatial mean s-MTF curve for the entire image. More recently, Cuccia et al.^4^^,^^5^ have developed spatial frequency domain imaging (SFDI) which employs single frequency sinusoidal patterns to extract single spatial frequency reflectance maps. The s-MTF is then fit to these maps pixel-by-pixel, resulting in images of absorption ($\mu_{a}$) and reduced scattering ($\mu_{s}^{\prime }$) coefficients.

The use of sinusoidal patterns in conventional SFDI presents a data acquisition bottleneck that limits SFDI imaging speed. Current SFDI instruments typically use digital micromirror devices (DMDs) to project spatially modulated light onto the sample. These DMDs are based on arrays of mirrors. Each of the array elements flickers on and off several times to generate grayscale intensities, resulting in pattern refresh rates typically in the order of hundreds of Hz. High-end scientific-grade CMOS cameras have the ability to acquire frames in the order of kHz or greater, exceeding the grayscale pattern refresh rate of DMDs. In many situations, such as those where the sample is susceptible to motion artifacts (e.g., clinical data acquisition) or high-temporal dynamics are required (e.g., neuroscience/cerebral hemodynamics),^6^^,^^7^ data acquisition speed is critical. Additionally, certain applications require multiple spatial frequency components, most notably SFD tomography, which relies on the spatial frequency dependence of depth penetration in turbid media.^8^^,^^9^ Ideally, multiple AC (nonplanar) spatial frequency components could be extracted from a sample simultaneously, although this is not possible using sinusoidal patterns. Square wave patterns contain frequency components at the harmonics of the fundamental frequency which can be synthesized into each SFDI frame, increasing the amount of spatial frequency information embedded into each frame of data.

The goal of this work is to increase SFDI data acquisition speed by an order of magnitude or greater by using binary, square wave patterns. For applications where higher AC (modulated) spatial frequencies are required, such as superficial tissue characterization and characterizing scattering contrast,^10^^–^^12^ square waves having a higher fundamental frequency can be used, such that the higher ordered terms exceed the s-MTF of the sample and are not present in the reflected light. In subsurface applications such as SFD tomography where multiple, relatively low-AC frequencies are required, square waves having a lower fundamental frequency can be used such that a subset of the harmonic terms are preserved.

In order to extract these additional harmonic terms, we have developed a multifrequency synthesis and extraction (MSE) algorithm. Here, custom patterns having known Fourier series coefficients are applied to the sample. The sample acts as a low-pass filter characterized by the sample’s s-MTF and attenuating the spatial frequency components of the diffusely reflected light. By changing the amplitude and phase of the Fourier series components of the pattern, a system of equations is established for each Fourier component at each pixel in the image, and can be solved using a pseudoinverse. MSE has the flexibility of extracting multiple frequency components from a sample assuming the base frequency and duty factor of the custom pattern are known, and the camera has high-enough dynamic range and signal-to-noise ratio to extract each harmonic component. In this case, we are using square wave patterns having discrete Fourier components at the harmonics of the square wave fundamental frequency.

We show agreement between results obtained using square wave and sinusoidal projection patterns by comparing $\mu_{a}$ and $\mu_{s}^{\prime }$ maps obtained from an *in vivo* human forearm. In our first experiment, we employ a high-frequency square wave pattern to extract DC (planar) and single AC (fundamental) spatial frequency components using both conventional SFDI demodulation and MSE. In the second experiment, we employ low-frequency square wave patterns to extract multiple spatial frequency components (DC, fundamental, and second harmonics) using MSE. Also shown are multifrequency depth penetration results on a phantom containing a buried absorbing tube surrounded by a turbid medium. Here, DC, fundamental, and second harmonic components are extracted from two low-frequency square wave patterns having different fundamental frequencies. We show agreement to within 1% of conventional SFDI for mapping $\mu_{a}$ and $\mu_{s}^{\prime }$ on an *in vivo* forearm, and determining reflectance versus depth on an absorbing tube phantom. Our results imply that SFDI data acquisition speed for mapping $\mu_{a}$ and $\mu_{s}^{\prime }$ and probing buried inclusions can be increased by an order of magnitude or greater with minimal losses in data quality using square wave patterns and MSE.

## 2 Materials and Methods

### 2.1 Spatial Frequency Domain Imaging

The SFDI workflow, including data acquisition, processing, and analysis have been previously reported.^5^ First, sinusoidal illumination patterns are projected onto a sample and a camera detects the remitted light, which has spatial frequency constituents that have been damped due to the absorption and scattering properties of the sample. In conventional SFDI, 2-D sinusoidal patterns having a single modulation frequency are projected sequentially at three distinct phases (0 deg, 120 deg, and 240 deg). A remitted light image is captured for each phase per spatial frequency. A simple demodulation equation based on square-law detection^4^^,^^13^ shown in Eq. (1) is applied to the three-phase detected light. Here, $M_{\mathrm{AC}}$, $M_{\mathrm{DC}}$, ω, and φ denote the demodulated AC and DC reflectance, angular frequency, and phase of the projected pattern, respectively. In this case, we assume the sinusoidal pattern varies in the horizontal (x) axis and stays constant along the vertical (y) axis.

$$M_{\mathrm{AC}}(x,\omega_{x})=\frac{2^{1/2}}{3}{\left\{{\left[I_{0\;\deg}(x)-I_{120\;\deg}(x)\right]}^{2}+{\left[I_{120\;\deg}(x)-I_{240\;\deg}(x)\right]}^{2}\;+{\left[I_{240\;\deg}(x)-I_{0\;\deg}(x)\right]}^{2}\right\}}^{1/2}\;I_{\varphi }(x,y)=\frac{M_{\mathrm{DC}}}{2}+\frac{M_{\mathrm{AC}}}{2}\cos(\omega_{x}+\varphi ).$$ (1)

The SFDI data collection process is repeated for a reference tissue simulating phantom having known optical properties. The data acquired from the reference phantom is used to normalize the sample intensity to account for the s-MTF of the SFDI instrument. Finally, the calibrated reflectance data at multiple spatial frequencies is used to derive the sample’s s-MTF, from which sample (unknown) optical property maps are determined.

Conventional SFDI uses sinusoidal patterns, thus spatial frequency data is sequentially acquired. Here we present MSE, a new method for extracting spatial frequency information content, thus capturing the s-MTF of turbid media, including biological tissue. To enable high-speed SFDI data acquisition, we make use of rapidly-generated binary square wave patterns containing multiple spatial frequency components.

### 2.2 Multifrequency Synthesis and Extraction Technique

The goal of MSE is to use custom patterns having multiple spatial frequency components, and extract the attenuated spatial frequency components remitted from the sample. First, multifrequency illumination patterns are projected onto a sample at different phases and a camera detects the remitted light. Each spatial frequency component in the custom pattern is simultaneously attenuated by the sample. We can express our series of raw intensity images as a vector (I), which is the product of the Fourier series coefficients of each frame with the reflectance at each spatial frequency component, shown in Eq. (2). Here, C represents the frequency amplitude and phase maps for each projected pattern (i.e., the Fourier coefficient matrix). For consistency, we express each frequency component as a real-valued sinusoid, although single complex exponentials (analytical expression) can also be used. R represents the amplitude attenuation for each frequency component in the reflectance maps. k and p are the indices for the Fourier component and projected pattern, and m and n are the total number of projected patterns and Fourier components, respectively.

$$I_{p}(x,y)=C_{k,p}(x,y)*R_{k}(x,y)\to R_{k}(x,y)=C_{p,k}{\left(x,y\right)}^{-1}*I_{p}(x,y),\;I_{p}(x,y)=[\begin{array}{c} \begin{array}{c} I_{1}(x,y) \end{array}\\ \vdots \\ I_{m}(x,y) \end{array}]\;R_{k}(x,y)=[\begin{array}{c} \begin{array}{c} R_{1}(x,y) \end{array}\\ \vdots \\ R_{n}(x,y) \end{array}],\;C_{p,k}(x,y)=[\begin{array}{ccc} C_{1,1}(x,y)\cos[\omega_{x,y,1}+\varphi_{1}(x,y)] & \ldots & C_{1,n}(x,y)\cos[\omega_{x,y,n}+\varphi_{1}(x,y)]\\ \vdots & \ddots & \vdots \\ C_{m,1}(x,y)\cos[\omega_{x,y,1}+\varphi_{m}(x,y)] & \cdots & C_{m,n}(x,y)\cos[\omega_{x,y,n}+\varphi_{m}(x,y)] \end{array}].$$ (2)

In principle, MSE can be applied to any multifrequency pattern, assuming that the phase and amplitude of each frequency component are known. Here, we are employing binary square wave patterns for the aforementioned projection speed benefit. In general, a square wave pattern in one dimension can be expressed by a Fourier series, shown in Eq. (3). Here, d is the duty cycle of the square wave, denoted as the fraction of high to low intensity values, and ω and φ are the angular frequency and phase, respectively. This is an infinite series, however, the low-pass filter nature of biological tissue eliminates higher-ordered harmonics, which can be neglected assuming they have been sufficiently damped (approximately an order of magnitude or greater) relative to the previous terms in the Fourier series This concept will be demonstrated in Sec. 3.

$$\text{Square wave}(x)=\frac{4}{\pi }\overset{\infty }{\underset{k=1}{\sum }}(\frac{1}{k})\sin(k\pi d)\cos(k\omega x+k\varphi )\;=\frac{4}{\pi }[\sin(\pi d)\cos(\omega x+\varphi )+\;\frac{\sin(2\pi d)}{2}\cos(2\omega x+2\varphi )+\frac{\sin(3\pi d)}{3}\cos(3\omega x+3\varphi )+\cdots ].$$ (3)

**Fig. 1:**
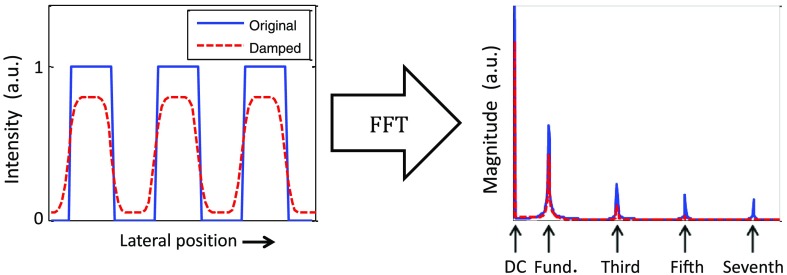
Simulation of one-dimensional cross-section of square wave pattern (50% duty cycle) interacting with turbid media. After interacting with a sample, the edge of the square wave is blurred in space. As a result, each frequency component is simultaneously attenuated.

Figure 1 illustrates a cross-section of a 2-D square wave pattern. The duty cycle of the pattern can be adjusted to change the Fourier coefficients of each harmonic component. After interacting with a sample, the edges of the pattern are blurred, thus the pattern appears more sinusoidal. In reality, a combination of multiple frequency components is embedded into the pattern.

To extract information from a given pattern using MSE, the amplitude and phase of each frequency component in the pattern must be determined. The amplitude coefficients are known from the analytical expression of the pattern itself. However, deriving the phase is nontrivial. For one, the position of the phase of the pattern field generated will most likely not match what the camera detects. For example, the camera requires a field of view that is smaller than the projected pattern,^14^ thus the field of view of the camera will not match that of the projected pattern. Second, depending on the angle of the camera relative to the projected patterns, sample topography will affect the phase angle.^15^ We previously, developed a technique using a variant of a 2-D Hilbert transform to express SFDI images in their analytical form, from which amplitude and phase angle maps can be determined.^16^ The phase maps generated using the Hilbert technique also adapt to surface topography. We have integrated the phase mapping capability of the Hilbert technique into MSE.

When low-frequency square wave patterns are used, the phase angle for each frequency component cannot be derived directly from the Hilbert technique. The reason for this is that the Hilbert technique relies on a single frequency spatial pattern. The fact that these low-frequency patterns contain multiple frequency components results in the mapping of a weighted sum of the phase angles, so separating the phases of different frequency components is not possible. To circumvent this issue, we employed a real-valued 2-D Morlet Wavelet filter using an open source toolbox (YAWTb: Yet Another Wavelet Toolbox) to isolate the fundamental frequency component from the pattern. For each raw image, the following steps are performed: (1) take a 2-D FFT of the image. (2) Multiply the image in the Fourier domain by the 2-D wavelet filter mask having a center frequency and bandwidth equal to the fundamental frequency of the square wave. (3) Take an inverse 2-D FFT on the image. (4) Apply the filtered image to the Hilbert phase angle mapping technique, using the mean of the raw images as a DC image for DC subtraction. (5) Apply the known Fourier series expansion of the pattern [from Eq. (3)] to the phase angle map to determine the amplitude and phase for each frequency component.

In Sec. 3, we demonstrate that $\mu_{a}$ and $\mu_{s}^{\prime }$ can be accurately determined on an *in vivo* forearm using a square wave pattern by extracting DC and fundamental frequency components. Also shown are the $\mu_{a}$ and $\mu_{s}^{\prime }$ results using a lower fundamental frequency square wave pattern, from which three spatial frequency components (DC, fundamental, and second harmonic) are extracted and used to determine $\mu_{a}$ and $\mu_{s}^{\prime }$. Finally, we exhibit the potential for MSE to extract data capable of layered or tomographic reconstruction by measuring reflectance versus depth using two square wave patterns having different fundamental frequencies, and extracting DC, fundamental, and second harmonic components from each.

## 3 Results

To generate the data used to produce the images analyzed in this section, we employed a second generation clinical SFDI system (VIS-NIR, Modulated Imaging Inc., Irvine, California). All data processing and computation used to produce figures was performed using the MATLAB^®^ software suite (MATLAB^®^ and Statistics Toolbox Release 2012b, The MathWorks, Inc., Natick, Massachusetts). Studies were carried out under the UC Irvine IRB approved protocol (HS# 2011-8370), and informed consent was obtained from all subjects.

### 3.1 Preliminary Experiments: Multifrequency Synthesis and Extraction Simulation and Tissue Phantom Reflectance

**Fig. 2:**
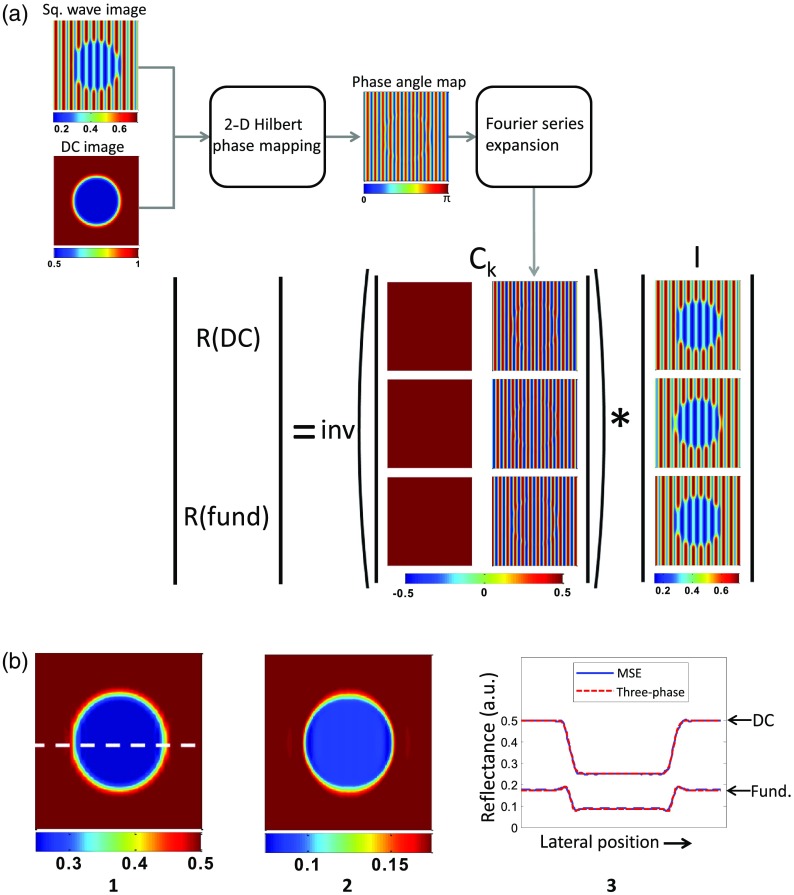
(a) Simulated workflow of multifrequency synthesis and extraction (MSE) algorithm on a turbid sample containing a circular absorbing lesion. First, a square wave (duty cycle=50%) and a DC (planar) image are acquired. Here, the low-pass filtering properties of the sample damp the higher-ordered harmonics of the square wave, such that only the fundamental frequency component is preserved. These images are applied to a two-dimensional Hilbert transform method, from which the phase angle map of the square wave pattern is derived. Using the known Fourier series representation of a square wave [Eq. (3)], the frequency coefficient matrix ($C_{k}$) is generated. Finally, $C_{k}$ is inverted and multiplied by the raw data vector (I). (b) Extracted spatial frequency intensities from simulation shown in (a), including (1) DC and (2) fundamental frequencies. (3) Cross-section of extracted reflectance comparing MSE to conventional, three-phase SFDI.

To demonstrate our new technique, Fig. 2 shows results on a simulated sample consisting of an absorbing lesion and a uniform scattering background. This is the simplest case of MSE, where the sample filters out all AC frequency components in the square wave except for the fundamental. A damped square wave image, which appears sinusoidal, and a DC image are applied to the Hilbert technique to extract a phase angle map. Next, phase map coefficients are derived by using the phase angle map in conjunction with the square wave Fourier series expansion. Finally, the Fourier coefficient matrix C is inverted and multiplied by the raw intensity vector I to obtain the reflectance vector R. For simplicity, Fig. 2 illustrates the MSE for a damped square wave pattern having harmonic components which surpass the s-MTF limit of the sample, such that only a single frequency component remains in the reflected light. However, MSE has the flexibility to extract multiple spatial frequency components from a pattern.

**Fig. 3:**
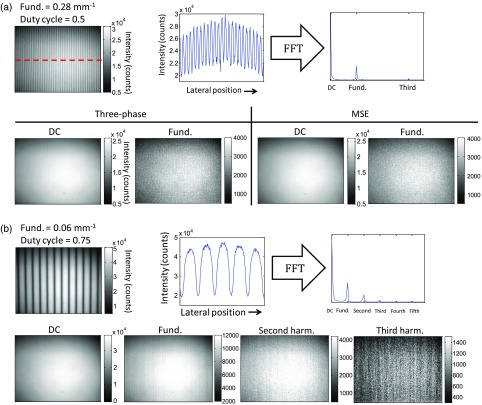
Square wave reflectance results on a tissue phantom having known optical properties ($\mu_{a}=0.0177$, $\mu_{s}^{\prime }=1.0023\;{\mathrm{mm}}^{-1}$ at $659\;{\mathrm{mm}}^{-1}$). (a) A high-frequency ($0.28\;{\mathrm{mm}}^{-1}$) square wave appears sinusoidal as the light is reflected from the phantom (top). In this case, the DC (planar) and fundamental frequency from the pattern are extracted. (b) The light reflected from a low frequency ($0.06\;{\mathrm{mm}}^{-1}$) pattern contains harmonic components, which are extracted using MSE. The third harmonic component is corrupted by noise.

In a preliminary experiment, we show reflectance data taken on a tissue-simulating phantom having known optical properties using square wave patterns having different base frequencies and duty cycles. These results are shown in Fig. 3. Here, we use both high and low fundamental frequency square waves to characterize spatial frequency component intensities of the reflected light in the phantom. Using the high-frequency pattern ($0.28\;{\mathrm{mm}}^{-1}$), only the fundamental frequency component is preserved. The reflected light from the low-frequency pattern ($0.06\;{\mathrm{mm}}^{-1}$), by comparison, contains harmonic components which are extracted. The third harmonic (fourth term) in this case is corrupted by noise.

Fitting to the s-MTF requires a minimum of two spatial frequencies, which are used to decouple $\mu_{s}^{\prime }$ from $\mu_{a}$. In the simplest case, we can employ a DC (planar) and a single AC (modulated) component to derive $\mu_{a}$ and $\mu_{s}^{\prime }$ maps, which is demonstrated in Sec. 3.2 using a square wave pattern at a relatively high-fundamental spatial frequency. In Sec. 3.3, we demonstrate how multiple AC frequency components can be extracted from a sample using a single square wave pattern at several phases having a relatively low-fundamental spatial frequency. We then use this data to map $\mu_{a}$ and $\mu_{s}^{\prime }$. In Sec. 3.4, we show reflectance maps taken from a phantom consisting of a buried tube containing an absorbing dye occupying a range of depths surrounded by a background of intralipid.

### 3.2 High-Spatial Frequency In Vivo Optical Property Extraction

**Fig. 4:**
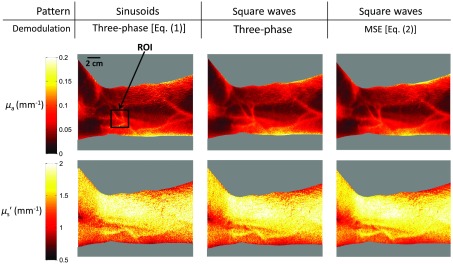
Absorption and reduced scattering ($\mu_{a}$ and $\mu_{s}^{\prime }$) maps generated using (left) sinusoidal patterns and three-phase demodulation (conventional SFDI), square wave patterns and (middle) three-phase demodulation and (right) MSE approaches, using three-phase-offset square wave patterns at a wavelength of 659 nm. Here we see agreement in $\mu_{a}$ values derived using square waves in the region of interest (ROI, black box) to within 0.5% and 0.3% for three-phase and MSE approaches, respectively. $\mu_{s}^{\prime }$ values show agreement to within 0.2% and 0.5% for three-phase and MSE approaches, respectively. For the entire field of view, the see average $\mu_{a}$ and $\mu_{s}^{\prime }$ values generated using square waves that agree to within 0.78% and 0.45% of conventional SFDI, respectively.

We performed a side-by-side comparison of optical property maps derived using two spatial frequency components extracted using conventional, three-phase demodulation Eq. (1) and MSE at a modulation frequency of $0.28\;{\mathrm{mm}}^{-1}$, and a source wavelength of 659 nm, shown in Fig. 4. For conventional SFDI, three-phase-offset sinusoidal patterns Eq. (1) and a DC frame are taken to extract the DC and AC spatial frequency components. For MSE, three-phase-offset square wave patterns with a duty cycle of 50% are taken. The 50% duty cycle was chosen to maximize the separation between the fundamental and nearest harmonic component to allow for optimal damping, since 50% duty cycle square waves have no even (i.e., second) harmonic components.

Figure 4 shows agreement in optical property values to within 1% for both $\mu_{a}$ and $\mu_{s}^{\prime }$ employing sinusoidal and square wave illumination using three-phase demodulation Eq. (1) and MSE approaches, respectively. These findings imply that, for cases where high-spatial frequencies are required, square wave patterns can be employed instead of sinusoids, assuming the harmonic components surpass the s-MTF of the sample. The reason for this is because the higher ordered terms in the square wave pattern (i.e., third, fifth, seventh, etc., harmonics) are highly attenuated relative to the fundamental term, and thus can be neglected in the MSE phase mapping and inversion algorithm. In this case, the conventional three-phase demodulation Eq. (1) can also be used, since the pattern appears sinusoidal, and thus contains a single AC frequency. In Fig. 4, we decouple the effects of the square wave damping from MSE by demonstrating that both three-phase demodulation and MSE are accurate in deriving $\mu_{a}$ and $\mu_{s}^{\prime }$ on the same dataset.

It should be noted that for square wave patterns to produce high-fidelity optical property maps, the choice of spatial frequency of illumination is nontrivial. In the case above, we chose a fundamental frequency such that the higher-ordered harmonics are essentially eliminated due to the filtering properties of the sample. We found that for most biological samples, a 50% duty cycle square wave with a fundamental frequency less than $0.25\;{\mathrm{mm}}^{-1}$ will generate higher ordered terms on a typical biological sample ($\mu_{a}=0.02\;{\mathrm{mm}}^{-1}$, $\mu_{s}^{\prime }=1\;{\mathrm{mm}}^{-1}$). In this case, the intensity of the next (third order) terms is roughly an order of magnitude less than the fundamental term. Additionally, since square wave patterns have only two unique intensity values (off or on), the number of projector pixels used to generate a single period of the pattern must be even (for 50% duty cycle), such that the duty cycle is consistent. The pixel length of the projected square wave period should also be divisible by the number of phases used in order to avoid duty cycle changes from phase-shifting the pattern.

Using high-spatial frequency ($>0.25\;{\mathrm{mm}}^{-1}$) square waves, the remitted light appears sinusoidal in biological tissue. Thus, high-frequency square wave patterns can be used to extract a single AC frequency component employing either three-phase demodulation or MSE. In this case, the same amount of information is acquired compared to sinusoids with 1 to 2 orders of magnitude faster projection speeds. To fully utilize MSE, the remitted light in the raw data images may contain multiple spatial frequency components, whereas the three-phase approach Eq. (1) relies on sinusoidal patterns (one AC spatial frequency component). We show that it is possible to extract multiple spatial frequency components from a square wave pattern in the next experiment using a pattern having a relatively low-fundamental frequency, such that multiple frequency components are preserved, and can thus be factored into the MSE inversion algorithm and extracted.

### 3.3 Low-Spatial Frequency In Vivo Optical Property Extraction

**Fig. 5:**
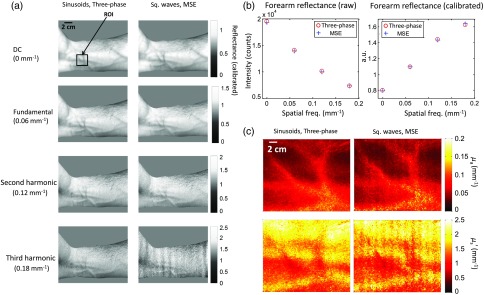
*In vivo* forearm results using square wave patterns and MSE. (a) Calibrated reflectance maps extracted at DC, fundamental ($0.06\;{\mathrm{mm}}^{-1}$), second, and third harmonic frequencies. The third harmonic image is corrupted by noise. (b) Mean raw and calibrated reflectance values from forearm ROI (black box) at DC, fundamental, second, and third harmonics. Calibrated reflectance values in the ROI agree with conventional SFDI to within 0.1%, 0.3%, 0.5%, and 4.2%, respectively. (c) $\mu_{a}$ and $\mu_{s}^{\prime }$ maps derived using diffusion model fit and DC, fundamental, and second harmonic reflectance maps. Mean $\mu_{a}$ and $\mu_{s}^{\prime }$ agree to within 0.5% and 1.0%, respectively.

In a similar manner to the previous experiment, we compared optical property maps derived using MSE to three-phase demodulation. In this case, we use a square wave pattern having a relatively low-spatial frequency, such that multiple frequency components are preserved. We extracted DC and three AC frequency spatial frequency components, and applied the DC, fundamental, and second harmonic components to a diffusion model to determine $\mu_{a}$ and $\mu_{s}^{\prime }$. Since we are using multiple AC frequencies, the accuracy of optical property fitting increases since there are more s-MTF points along which to fit. These results are shown in Fig. 5, where seven uniformly phase-offset square wave patterns having a fundamental frequency of $0.06\;{\mathrm{mm}}^{-1}$ and a duty cycle of 75% are used to extract the DC, fundamental, second, and third harmonic components, corresponding to 0, 0.06, 0.12, and $0.18\;{\mathrm{mm}}^{-1}$, respectively. The 0, 0.06, and $0.12\;{\mathrm{mm}}^{-1}$ component maps are applied to a diffusion model from which $\mu_{a}$ and $\mu_{s}^{\prime }$ maps are derived. These optical property values are compared directly to those obtained using three-phase demodulation and sinusoidal patterns at the same spatial frequencies.

Figure 5 illustrates that higher ordered harmonics can be extracted using a single multifrequency square wave pattern, and that $\mu_{a}$ and $\mu_{s}^{\prime }$ values agree with those obtained using conventional, three-phase demodulation and single-frequency sinusoidal patterns using a diffusion model. The use of multiple AC spatial frequency components increases the accuracy of optical property mapping, and thus the quantitation of chromophore concentrations and structural parameters.

It should be noted that the presence of noise related to higher-ordered harmonics increases with the higher-ordered terms (i.e., third harmonic in this case). This is due to the fact that the higher-ordered terms in a square wave pattern have less intensity relative to the lower ordered terms. In Fig. 5, we calculate optical properties using the DC, fundamental, and second harmonic terms, and discard the third harmonic term, which is corrupted by noise.

As mentioned in Sec. 1, the mean interrogation depth of SFDI patterns in turbid media is dependent on the spatial frequency component; lower spatial frequencies penetrate deeper while higher spatial frequencies probe more superficial layers. Thus, SFD tomography is possible by extracting and analyzing multiple spatial frequency components. For these tomographic reconstructions to be accurate, each spatial frequency component in a given pattern must interrogate the appropriate depth. In the following section, we present results obtained from a tissue simulating phantom having a buried tube containing an absorbing dye. The results show that square wave patterns applied to MSE yield reflectance maps similar to those obtained using three-phase SFDI within a simple tomographic context.

### 3.4 Depth Penetration Experiment Using Buried Absorber Phantom

**Fig. 6:**
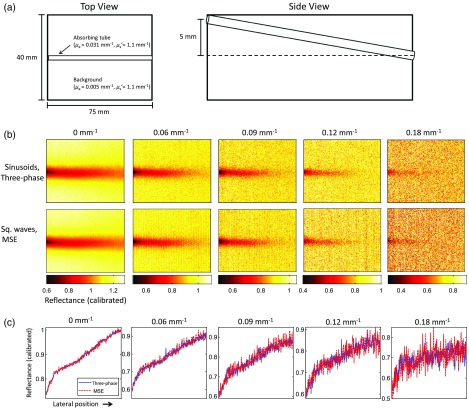
Multispatial frequency reflectance results obtained on a phantom containing a slanted absorbing tube ranging in depth from 0 to 5 mm, containing an absorbing dye with a scattering background. (a) Schematic of phantom geometry. (b) Reflectance maps calibrated to a homogenous tissue-simulating phantom are derived for DC ($0\;{\mathrm{mm}}^{-1}$), 0.06, 0.09, 0.12, and $0.18\;{\mathrm{mm}}^{-1}$ using the fundamental and second harmonic components from two square wave patterns. (c) Cross-sections of calibrated reflectance taken from horizontal line in center of image where tube is located. Mean reflectance values along the line agree to within 0.04%, 0.1%, 0.4%, 0.1%, and 0.12% for 0, 0.06, 0.09, 0.12, and $0.18\;{\mathrm{mm}}^{-1}$ respectively.

Multiple SFDI spatial frequency components can be extracted and processed to perform three-dimensional reconstructions of buried absorbers.^8^^,^^9^ To test our ability to extract the correct depth information using square wave patterns, we applied relatively low-spatial frequency square wave patterns (0.06 and $0.09\;{\mathrm{mm}}^{-1}$), with a duty cycle of 75%, utilizing the DC, fundamental, and second harmonic spatial frequency components, to a tissue phantom containing a buried absorbing tube oriented diagonally in depth, such that the depth of the tube ranged continuously from 0 to 5 mm beneath the surface. The tube contained a solution of 0.5  g/L of an NIR absorbing dye (NIR746A, QCR Solutions Corp., Fort St. Lucie, Florida). This formulation was chosen such that the $\mu_{a}$ of the tube mimicked venous blood at 731 nm. The background contained a 1% solution of intralipid, which has a $\mu_{s}^{\prime }$ comparable to human skin.^17^^,^^18^ Figure 6 shows results comparing reflectance maps taken at 731 nm using sinusoidal patterns combined with three-phase demodulation, and square wave patterns combined with MSE.

The results shown in Fig. 6 indicate that the multispatial frequency components extracted using MSE and square wave patterns yield depth penetration reflectance similar to three-phase demodulation using single frequency patterns for DC, fundamental, and second harmonic components. This implies that MSE and square wave patterns can be used to extract multifrequency datasets used in SFD tomography.

## 4 Discussion

SFDI has the ability to provide information-rich datasets based on the acquisition and analysis of spatial frequency domain reflectance maps, allowing for quantitative analysis of biological tissue. SFDI data should be acquired as quickly as possible to enable the visualization of dynamic signals and mitigate artifacts related to motion. To maximize data acquisition speed, frame rates should be limited to the camera exposure time. Sinusoidal projection patterns used in conventional SFDI take significantly longer to project than the exposure times of most high-end, scientific-grade cameras, resulting in a data acquisition speed bottleneck. This work introduces the combined use of a new signal processing technique (MSE) and the use of square wave projection patterns, which can be generated faster than the frame rates of current high-end cameras.

Our results demonstrate that $\mu_{a}$ and $\mu_{s}^{\prime }$ maps derived using MSE and square wave patterns are essentially identical to those derived from conventional SFDI. In Sec. 3.2, we employed a square wave pattern with higher ordered harmonic components that exceed the s-MTF limit of a biological sample, a configuration suitable for cases where sensitivity to superficial tissue layers or scattering is emphasized. Here, the fundamental component is left intact, and the reflectance information contained in this pattern is extracted. We obtained $\mu_{a}$ and $\mu_{s}^{\prime }$ maps with agreement to within 1% of conventional SFDI. In Sec. 3.3, we used a square wave pattern having a relatively low fundamental spatial frequency, such that the fundamental and harmonic components are left intact. This configuration is suitable for cases where probing deeper tissue layers or s-MTF fitting accuracy is emphasized. Here, we derived reflectance maps at DC, fundamental, and second harmonics, and computed $\mu_{a}$ and $\mu_{s}^{\prime }$ that agree to within 1% of those derived using conventional SFDI. The requirement for optical property fitting accuracy will depend on the chromophores or structural parameters of interest and the application. However, we expect that a 1% margin of error for $\mu_{a}$ and $\mu_{s}^{\prime }$ will be acceptable for most cases.

In Sec. 3.4, we demonstrated the potential for MSE and square wave patterns to generate SFD tomography datasets. We applied two square wave patterns having relatively low fundamental frequencies to a phantom containing a buried, slanted absorbing tube having a continuum of absorber depths as a function of lateral spatial location. Here, reflectance values obtained along the tube at multiple spatial frequencies using MSE and square wave patterns agree to within 1% of conventional SFDI. Importantly, our ability to control the optical path length with spatial frequency is clearly evident as the deeper portions of the tube disappear from view at higher spatial frequencies.

A single snapshot optical properties method (SSOP) has previously been developed which employs a single sinusoidal pattern to map optical properties, reducing frame count over a conventional SFDI.^6^ Here, a 1-D Fourier transform is applied to each row or column in the intensity image, and the spectrum is separated into DC and AC components. Although this technique reduces data acquisition time by using fewer frames, the approach is currently limited to single-frequency sinusoidal patterns, resulting in limited pattern refresh rates and spatial frequency information content. It may be possible to combine square wave patterns with SSOP processing to allow for increased pattern projection rates, as well as multifrequency extraction using a single snapshot.

As mentioned in Sec. 2, the Hilbert demodulation technique used in MSE phase angle mapping has the ability to adapt to pattern distortions due to surface topography. Thus, it should be possible to use Hilbert demodulation to acquire surface profilometry data. In particular, a single, high-frequency square wave pattern whose pattern orientation is orthogonal to the plane formed by the camera and projector optical axis^15^ could be used to determine surface topography. This combined Hilbert and square wave approach would be much faster than the conventional method, which uses three phase-offset sinusoidal patterns to determine phase angle.

Our eventual goal is to both acquire and process SFDI data in real-time, and processing requires computation time. In the case of three-phase demodulation Eq. (1), the mathematical operators have linear or near linear computational complexities, denoted in Big O notation by O(n), where n represents the number of digits used for each pixel, or simply each pixel, since the operation is linear. MSE, on the other hand, consists of a pseudo-inverse and matrix multiplication, which have computational complexities of approximately $O(n^{3})$ and O(nmp), respectively, where n, m, and p represent the number of projections, number of spatial frequencies, and 1 (columns in I matrix), respectively. In the case of the multifrequency experiments where seven projection patterns are used and three AC spatial frequencies are extracted, this translates to roughly 124 linear operations per pixel per AC spatial frequency. By comparison, the three-phase equation consists of 10 linear operations for each pixel. Consequently, MSE is expected to have approximately an order of magnitude more computational demand than the three-phase equation. Advanced techniques such as parallel processing on graphics processing units could be used to alleviate this increased computation burden.

MSE can accommodate projection patterns having arbitrary spatial frequency content. Thus, there is potential for the use of alternate spatial light modulators (SLMs). A rotating fan, e.g., contains radially-varying square wave patterns. Such a device would be far less costly than a DMD and would have no refresh rate. Alternatively, a light source having intrinsic spatial frequency patterns such as an LED array could be employed, eliminating the need for an SLM, potentially reducing the footprint and complexity of SFDI instruments.

## 5 Conclusion

We have described and demonstrated a new algorithm (MSE) for extracting images of multiple spatial frequency components using square wave patterns of structured light. By using square wave patterns, SFDI data acquisition speed is potentially increased by an order of magnitude or greater. We have applied MSE to an *in vivo* forearm model and a tissue-simulating phantom that confirms both the accuracy of optical property reconstructions and the depth sensitivity of the technique. The use of binary patterns and MSE in the SFDI workflow will ultimately enable the development of a video-rate SFDI instrument with multispectral and multifrequency capability.
